# Medical and Philosophical Intelligence

**Published:** 1812-04

**Authors:** 


### MEDICAL AND PHILOSOPHICAL INTELLIGENCE.

### MEDICAL SOCIETY OF LONDON.

AT a quarter before four o'clock, on Monday, March 9, the usual annual oration was delivered by Dr. Temple. He began by exhibiting a brief and succinct view of the various theories that have prevailed in physic, from the earliest ages in which medicine was studied and taught as a science, to the conclusion of the eighteenth century.

The

The doctrines of Hippocrates, Democritus, Asclepiades, and Aretæus, subsequently reduced to order and method by Galen, and from him continued downwards for many centuries, passed successively in review before him. The appearance of the chemical sect, under Paracelsus, in the beginning of the sixteenth century, laid the foundation of an entirely new system, in direct opposition to that of Galen: these continued to divide the medical world, till both were in a great measure overthrown by the new lights of anatomy and physiology, and especially by the discovery of the circulation of the blood, about the middle of the seventeenth century. This discovery, however, great as it was, introduced a mechanical theory into physic, which, though more intelligible, as appealing more to the senses, was yet extremely defective, in overlooking the great influence of the living principle in every part of the animal economy. It is to Hoffman and Willis that we are principally indebted for first pointing out to us this the only proper path towards a pathology founded on just notions of the principle of life, as the efficient agent in all vital phenomena. While, however, the unfounded notions of the Galenical as well as the chemical sects were thus discarded by the more rational and philosophical views of Hoffman and his successors, the theory of the most important diseases is even yet unsettled, and far removed from certainty. Hypothesis has succeeded hypothesis, every one of which has had an unfavorable influence on the treatment of diseases.

From such a view of the actual state of medical doctrines, the orator deduced the necessity of confining our attention more closely to experience and observation, as the only safe guides; and he thought he could not better subserve the views of this society, on the present occasion, than by stating the result of his own experience regarding the use of certain articles of the materia medica, in the treatment of some of the most frequent and most important diseases.

The first subject on which he expatiated was rheumatism, which, in his opinion, was uselessly and improperly divided into two species, namely, the acute and chronic: for he had found in practice that the treatment of both was precisely or essentially the same. He condemned blood-letting in the acute rheumatism, as it is termed, however much this evacuation may seem to be indicated by the state of the pulse, and other symptoms; as it tends, according to his observation, to prolong the disease to an almost indefinite period, while it seldom gives more than very temporary and partial relief. Purgatives in the beginning he found almost universally useful; but the remedy upon which he principally and almost solely relied, was the oil of turpentine, in doses of 25 or 30 drops, every four hours, in 2 ounces of the decoction of Peruvian bark. This, he said, in the space of 24 hours, procured an abatement of pain and the other symptoms, and in a very few days, in almost every instance, effected a complete recovery.

Pleurisy was another disease that engaged his attention; and here he endeavored to show, that, after blood-letting had been practised to a certain extent, the cure might be safely trusted to digitalis, in doses, however, considerably beyond those that are ordinarily exhibited; namely, to the extent of 30 drops of the tincture of that plant every four hours. By the effect of this remedy in reducing the vascular action of the system, much loss of blood, he found, might be spared, and consequently the ultimate restoration of the patient to health and strength materially accelerated.

On the subject of dropsy, the orator remarked that he had found no remedy in anasarca at all equal to the supertartrite of potass, exhibited largely. It had the effect, in the greater number of instances, of discharging water copiously both by stool and urine. In ascites, the elaterium appeared to him to be the most powerful remedy, administered in the dose of a grain or two, about twice in a week, employing the usual tonic remedies on the intermediate days.

After a number of other observations of a strictly practical kind, the orator concluded with recommending to the younger part of his auditory the sedulous observation of diseases, and the effects of remedies in actual practice, as the surest and safest guide to the successful treatment of them.

---

Royal Society.—*Feb. 6th.* Dr. Wollaston read a paper on the primitive form of calcareous, bitter, and iron, spar. The conclusion Dr. Wollaston made from his observations, was, that a difference in constituent principles, produces, in every case, a difference in primitive form; that the exceptions to this general position are entirely owing to errors in our crystallographical operations; and that the crystallogical theory is strictly true to nature.

*Feb. 13th.* Mr. John Davy communicated by his brother, Dr. Davy, a paper in opposition to the opinion of M. M. Gay-Lussac, Thenard, and Murray, that there is no action between gaseous oxide of carbon and oxymuriatic gas, or *chlorine* (a name given to it from its green color), when exposed to the rays of the sun. Mr. Davy states a different result. He gives, likewise, an account of the nature and combinations of this substance, which neutralises four times its volume of ammonia, and forms with it a peculiar salt.

*Feb. 20th.* A paper on the combinations of certain metals with chlorine and oxygene, was communicated by Mr. John Davy. The pure combinations of metals with chlorine resemble in many respects oxides; they are non-conductors of electricity, many of them are very volatile, and they form muriates by decomposing water. The author describes two combinations of copper with chlorine, two with iron, and two with tin. Bismuth, arsenic, antimony, zinc, and lead, he has also been able to combine with chlorine. The experiments of Mr. J. Davy confirm the fact that oxymuriatic gas, or chlorine, has not yet been decompounded.

---

*Geological Society* held its general annual meeting on the 7th of February for electing its officers and council for the ensuing year.

---

*London Philosophical Society.*—A society which professes to hold principles and pursue objects which are those of every man who loves loves improvement, cannot pass unnoticed. The views of this society are fully explained by the following passage from its prospectus:—"To foster genius, to eradicate unphilosophical prejudice, to increase the knowledge of nature, and most of man; to destroy, as much as possible, that false definition of words which has been justly reprobated by Bacon and Locke as the origin of sophistry and misconception; but above all, to remove that barrier erected by pedantry against universal knowledge, which has introduced an *esprit de corps* into philosophy, and rendered it a sect rather than the province of the world." This is a consummation devoutly to be wished. The means it proposed to employ are lectures on every department of philosophy, which are to be subjected to a rigorous subsequent examination, in which truth is to be the object, and the inductive system of Bacon the portal to her shrine. The attention of the society has, in the preceding month, been directed to a brief course of Lectures on the Pyramids, by Mr. Clarkson, as preliminary to a regular attempt at illustrating the hieroglyphic language.

---

*Museum of Natural History.*—We have frequently had the opportunity to remark the industry, ingenuity, and talent, of Mr. Bullock, in the preservation and arrangement of the subjects of natural history; but we did not expect to see an individual, whose principal resources have arisen out of his own exertions, accomplish so much. The Museum in Piccadilly, which was, a few days since, opened to the public, presents, in the extensive collection and rare specimens of all the Linnæan classes, an opportunity to the scientific naturalist to pursue his favorite study without fatigue or danger. To the general admirer of Nature, whose views do not extend to the science, it will afford gratifications little less poignant. But, if the extensive collection of specimens tends to perfect and diffuse a knowledge of nature, the disposition of the building, and the arrangement of the subjects, will no less excite admiration. To remedy the imperfect manner in which collections of preserved quadrupeds, &c. especially the larger kinds, have hitherto been exhibited, and to give a verisimilitude to the scene, has been the object of Mr. Bullock. To effect this, the animals are shewn in the manner they would appear in their native wilds, keeping to the scientific arrangement of Linnæus. A considerable apartment, for this purpose, has been erected on a plan entirely new. The spectator, on entering this novel and interesting scene, finds himself suddenly transported into the centre of a tropical forest, in which, in appropriate situations, are seen most of the known quadrupeds from every part of the world, as well as correct models, from the best authorities, of the exotic trees and vegetable productions, most remarkable for their singularity, the luxuriance of their fruit, their foliage, or their uses in civilized society. The appearance of distance is given by a panoramic painting, and the illusion is so strong, that it is with difficulty the visitor can convince himself of his entire safety, and that he still is in the centre of a crowded metropolis. The blue hills hills and distant misty vallies are caught with a sort of magical effect, between the boles of the cocoa, the bread-fruit tree, &c. &c. The ornithological department is enlarged to double its former extent, and a considerable and fine collection of shells and other marine productions, fishes, &c. has been added.

Taken with its additions, the taste and novelty of the arrangement, the correct Egyptian style of architecture of the building, composed from authentic documents, this Museum presents a *tout ensemble* unique in its kind, forming the most extensive and interesting collection of natural history, perhaps, in the world.

---

Jacob Zink, of Glave Road, Mile End, chemist, has taken out a patent for a new method of manufacturing verdigris, which he denominates British verdigris.

---

Mr. Saumerez will shortly publish a work on the Philosophy of Physiology and Physics; comprehending an examination of the modern systems of philosophy.

---

In a former number we mentioned the proposal for establishing sea-water baths on an extensive scale in the neighbourhood of London; we have since received a more detailed account, which we now insert.

"In a country where the sources of rational amusement and social enjoyment flow uninterrupted by the calamities of war, and at a period when every improvement tending to promote health, or contribute to the comforts and happiness of society, is patronised with liberality; it is a matter of no small degree of surprise, that the metropolis should be unprovided with one of the most salutary establishments which art can devise, or to which nature can contribute.

"Amongst the auxiliaries which tend to promote health and longevity, that of bathing has, from the earliest ages to the present day, been admitted to be, and undoubtedly is, the most prominent. It is a fact well established, that public baths, on an extensive scale in the vicinity of London, have long been anxiously sought after, and still remain desiderata of the first consequence to its immense and increasing population; which, including the environs, amounts to 1,200,000. Of this number, four-fifths are, by various circumstances, debarred from the pleasure of a country excursion, or the benefit of an annual visit to the coast. Hence the origin of numerous diseases, especially of inveterate scrofulous and cutaneous distempers, which deform the person, and baffle the efforts of medical art.

"An eminent popular physician, the late Dr. Buchan, has observed, that there are few families in this country which have not a taint of scrofula. The forms in which it appears are numerous, but the most formidable one is that of pulmonary consumption; from which disease alone, according to the calculations made by the ingenious genious and scientific Dr. George Pearson, and communicated by him to the Royal Society, 'no less than from 120,000 to 140,000 fall victims annually in the British empire, in the prime and vigor of youth, and of this number at least one-fourth is confined to the metropolis and its environs.' If the other species of this malady are less fatal, they inflict much distress by affecting the joints with lameness, and disfiguring the body in various ways, to which death itself would be preferable.

"But the limits of this address will not admit the details of all the alarming evils which the inhabitants of this metropolis suffer from the different cutaneous, constitutional, and chronic, disorders, to which they are subjected in consequence of having no certain or appropriate means of *prevention*, as well as relief. It has long since been acknowledged by the most eminent physicians, that in these cases no remedies possess such specific powers as the sea-water bath, which conduces alike to comfort, luxury, and health; where it does not prove a complete remedy, it seldom fails to suspend the progress of the malady, and alleviate the sufferings of the patient.

"Dr. Willan, whose attention for many years was almost exclusively directed to cutaneous affections, has very justly remarked, 'It is singular that London, the most populous city of Europe, should not be provided with any extensive accommodation for bathing. Till this defect be remedied, its inhabitants must undergo many inconveniences, and be liable to many disorders, which are unknown in warm climates, where the use of baths has been at all times indispensably necessary. In the principal towns of Greece and Italy, there were Balnea, or public baths, of great extent. Ancient physicians considered them to be of so much importance for the preservation of health, that they have carefully transmitted to us a series of observations respecting their utility, and the mode of their application. Great advantage might likewise be derived from baths in our climate; it is therefore to be wished that they were more general, and of more easy access to the lower class of people: *the frequent use of them would not only relieve or prevent cutaneous complaints, but would be useful in a variety of internal diseases.*'

"A circumstance which further shews the imperious necessity of establishing large public baths is, that numerous persons daily subject themselves to the penalties of the law, by bathing in the New River and other waters proscribed by statute, rather than be deprived of the opportunity of immersion.

"The present prospectus is intended to develop the general outlines of a plan for establishing sea-water baths for the convenience of the metropolis and its environs. In order to obtain them on an extensive scale, it will be necessary to lay pipes (forming a main) from the coast of Essex to Copenhagen-fields, or some such elevated and eligible situation; where a reservoir covering several acres of land will be formed, into which, by means of a powerful steam engine, a constant supply of sea-water will be continually kept flowing. From this very extensive reservoir, the numerous baths would be supplied with a running stream. Engineers of the first eminence have sanctioned tioned the undertaking, and determined that it is completely practicable.

"It is also proposed to connect with this establishment every species of warm, cold, shower, and vapour, baths; and the spacious grounds, which will include a botanic garden, are to be laid out in tasteful walks, shrubberies, and lawns, forming splendid promenades, uniting an elegant resort, conducive equally to the purposes of health and rational amusement. A laboratory for preparing artificial mineral waters, and the gases used in pneumatic medicine, with apparatus for inhaling the same, will also be provided.

"A large and commodious hotel, judiciously fitted up with numerous apartments, as well as other buildings for libraries, &c. such as are deemed requisite at other bathing places, will form a part of this establishment; and it is intended that the whole shall be under the direction of a highly respectable committee, who will enforce the strictest regulations of decorum and good management.

"A very important feature of this undertaking, is the opportunity that will be afforded for establishing an extensive *Infirmary for Sea-water Baths.* All the afflictions incident to the human frame, over which medicine or surgery can prevail, have their separate establishments; and each disease has medical and surgical aid administered to it, by the proverbial bounty of our countrymen. Those diseases alone, which are beyond the power of medicine, and that are remediable only by sea-water bathing, have been left wholly destitute of asylum or relief, with the exception of the Margate Sea-bathing Infirmary; and that establishment, in consequence of its distance from the metropolis, and its limited funds, although unquestionably of much public benefit, is totally inadequate to contribute relief to the sufferings of thousands who are confined by penury, or business, to a constant residence in the metropolis.

"An important benefit will be attained in the arrangement of these baths, as unprecedented as it will be salutary, to those who frequent them; for the constant flow of sea-water proposed to be secured, must obviate the evils attendant on several persons laving in the same water, wherein an individual afflicted with an infectious disorder had previously bathed; a circumstance always heretofore dreaded, and very likely to occur in establishments of this nature on a limited scale, where a constant stream could not possibly be obtained.

"This outline of a scheme for effecting these great national improvements, is therefore, after due deliberation, submitted to the approbation of a British public, with entire confidence in its patronage of a plan which embraces objects of such vital importance to the community, and promises in a considerable degree to counteract the accumulating evils of an excessive population."

---

Mr. PURSH, of New York, is now in London preparing for the press a *Prodromus Floræ borealis Americanæ.* He is engaged in consulting the most celebrated libraries in this city, and expects, with his own extensive collections in conjunction with those ample resources, to succeed in giving a more perfect view of the Flora of

North America than heretofore has been published. A number of plants, not described in other works, will have their full elucidations, and some of the most interesting will be figured. Having himself paid considerable attention to the cultivation of plants, particularly as it regards climate and soil, he is enabled, in this Prodromus, to furnish many useful hints and directions to those who wish to grow the favorite plants of the American continent. The whole will be comprised in an 8vo. volume; and all such plants which the Prodromus cannot refer to good plates, will be from time to time published in a Flora, to follow the Prodromus as soon as the necessary arrangements can be made. From the opportunities which Mr. Pursh has had of becoming acquainted with the plants of North America, from his great industry, and his extensive and correct acquaintance with the subject, we do not doubt but his intended publication will be highly serviceable to botanical science.

---

Professor Leslie, of Edinburgh, has in the press a View of the Facts ascertained concerning Heat, and its relations with air and moisture.

---

*Case of Puerperal Convulsions, cured by very copious Blood-letting.*—A delicate little woman, eighteen years of age, a native of Halifax, in Nova Scotia, but residing with her husband in England, was in the seventh month of her pregnancy, when she was attacked early in the morning of the 17th of July, 1811, with puerperal convulsions. When visited by her medical attendant, she was lying in bed, in a comatose state, the pupils were contracted and immoveable, the pulse was above ninety, and not deficient in power. A few minutes after he entered the chamber, the convulsions returned again, the whole body was agitated by violent starts, the eyes were rolled about and drawn up under the lids, the mouth was drawn sometimes toward one ear and sometimes toward the other, the breath was sucked in with a hissing noise, and the whole countenance, which, when composed was pleasing and interesting, was distorted into the most frightful deformity. As soon as the convulsion was at an end, a basin full of blood, measuring twenty ounces, was taken from the arm, the pupils dilated, and she soon became sensible, which she had not been for several hours.

She was visited again in the middle of the day, in consequence of a hurried message. After the bleeding in the morning, she had remained sensible and free from convulsions for an hour, but, at the end of that time, she again became comatose, and again the convulsions returned. The bandage had slipped from the arm, and she lost some blood about the bed before the affrighted relation had coolness to tie up the wound again. The os uteri was slightly open; notwithstanding the quantity of blood that had been lost, the effect of the morning's blood-letting induced a repetition of it, the basin measuring twenty ounces was again filled to the brim; the medical attendant remaining in the house an hour, to watch the effect of the remedy. She continued comatose, but the convulsions became obviously less frequent and less violent.

She was visited again at ten o'clock at night. From the middle of the day to nearly that time, she had remained in the same state in which she had been left; but, a few minutes before the third visit, she had had several convulsions, quickly succeeding one another, and more violent than any former ones. An attempt was made to open the temporal artery, just before the ear, but it was so small, that but little blood could be procured from it. A vein was opened in each arm, and thirty ounces of blood were taken away. This, with the former bleedings, amounted to seventy ounces of blood, during thirteen hours. From this time the convulsions entirely subsided. She continued comatose during the remainder of the night, with only momentary intervals of consciousness, but towards the morning she awoke entirely out of her insensibility: about the middle of the second day she had some uterine pains, which grew stronger as night approached, and about one the next morning expelled a fœtus, which presented by the feet. For several days she was feverish, and slightly delirious; but these symptoms soon vanished, and a few weeks afterwards she left the neighbourhood entirely well. Although she had no complaint just before her attack, she has lost all memory of the previous fortnight.—*Lond. Med. Rev.*

---

Mr. Hogben, surgeon, of Berners-street, has nearly ready for the press, in two classes, a series of Anatomico-mechanical Tables of the Human Gravid Uterus, the size of life, and rendered more agreeable to Nature, than ever before appeared on paper. They represent parturition in its several stages, the various presentations of the head of the fœtus, surrounded by the distended uterus, in that clear and comprehensive manner necessary to guide the young accoucheur. The bones and sutures of the infant cranium; the infant pelvis, as at the day of birth; and adult pelves, variously formed; a section of the pelvis with its contents in the unimpregnated state, and every part of the bones which compose that part, are particularly described. The decidua, and decidua-reflexa, rendered singularly conspicuous; and all the essential parts connected with the science of midwifery clearly delineated.

Although composed of paper, and laid perfectly flat, yet they are so contrived that the real forceps may be applied to give a true representation of its proper application in a living subject, which it is presumed may prove useful to the young practitioner, as well as the student attending lectures; and the whole not uninteresting to all medical men.

Likewise an illustration of the Vectis; a view of the Fœtus in Utero, through the transparent membranes, surrounded by the liquor amnii; the uterine blood vessels, and the membranes as they appear when distending the os uteri, at the time they commonly rupture.

The numerous plates which compose this work, have been under the hands of several eminent engravers nearly four years, the whole of which is, by way of illustrating a Treatise on Parturition and the Science of Midwifery in general, explaining the different cases represented in the tables, showing the changeable stages of the fœtus during labor, demonstrating the application and different directions of the forceps as the head of the child descends from out of the uterus and vagina, by a kind of natural mechanism.

MONTHLY

